# Endotension After Endovascular Aneurysm Repair: The Critical Impact of Age and Reintervention on Survival

**DOI:** 10.7759/cureus.94398

**Published:** 2025-10-12

**Authors:** Mahmoud Bakheet, Yasser Elsayed, Selina Kwong Chian, Samuel Rhodes, Thamer Babiker, Mohamed Banihani

**Affiliations:** 1 Vascular Surgery, Royal Preston Hospital, Preston, GBR; 2 Vascular Surgery, Lancashire Teaching Hospitals NHS Foundation Trust, Preston, GBR

**Keywords:** anticoagulation, endotension, endovascular aneurysm repair, evar, reintervention, relining, rupture, type v endoleak

## Abstract

Objective: Endotension (type V endoleak), a condition of continued aneurysm sac expansion without a detectable endoleak after endovascular aneurysm repair (EVAR), remains a management challenge. This study analyzes the long-term outcomes, management strategies, and risk factors for rupture in patients with endotension.

Methods: A retrospective single-center analysis of 19 patients with endotension was conducted. Patients were stratified into two management groups: conservative (n = 11) and reintervention (n = 8). Data on aneurysm characteristics, sac dynamics, interventions, and outcomes were analyzed.

Results: The reintervention group was significantly younger than the conservative group (mean = 73.8 vs. 82.1 years, p = 0.009). The overall rupture rate was 26.3% (5/19). The conservative management group, despite comprising patients deemed unfit for intervention, had a 27.3% (3/11) rupture rate. In contrast, the reintervention group, treated primarily with relining (7/8), had a 25% (2/8) rupture rate; both cases were successfully treated emergently with 100% survival. Technical success for reintervention was 100%. Overall mortality was 36.8% (7/19). Sac progression stabilized in 40% (2/5) of anticoagulated patients following cessation of therapy.

Conclusions: Endotension carries a significant rupture risk, even under conservative management. A strategy of vigilant surveillance with a low threshold for reintervention in suitable candidates is critical. Endovascular relining is a highly effective and durable solution, even in emergencies.

## Introduction

Endovascular aneurysm repair (EVAR) has become the established first-line treatment for anatomically suitable abdominal aortic aneurysms (AAAs), offering reduced perioperative morbidity and mortality compared to open repair [1,2]. However, its long-term success is contingent on diligent surveillance to identify and manage complications, including endoleak, device migration, and aneurysm sac expansion [3,4].

Among these, endotension, a condition of persistent sac expansion without a radiologically demonstrable endoleak (type V endoleak), remains one of the most enigmatic and management-intensive complications [5,6]. First described over two decades ago, its pathophysiology is still not fully elucidated, with proposed mechanisms including pressure transmission through thrombus, ultrafiltration of blood, occult endoleaks below imaging resolution, or graft material permeability [7,8]. This diagnostic uncertainty creates a significant clinical dilemma: when to intervene on an expanding sac with no clear target, and what intervention strategy to select [9].

The natural history of endotension is poorly defined. While some aneurysms may stabilize, others progress relentlessly, culminating in rupture with potentially catastrophic consequences [10]. Current guidelines provide limited specific direction for endotension, leading to practice patterns that range from vigilant surveillance to pre-emptive reintervention [11,12].

Crucially, there remains a critical lack of robust, long-term data comparing the outcomes of conservative management versus reintervention for this condition. The factors that dictate management choice, particularly patient fitness, and the subsequent rupture risk under surveillance, are not well quantified [13,14].

This study aims to address this gap by presenting a decade of experience from a regional vascular center. We evaluated the natural progression, reintervention strategies, and survival outcomes for patients with endotension. Furthermore, we performed a comparative analysis of outcomes between patients managed conservatively and those who underwent reintervention, with the goal of informing clinical decision-making for this complex patient group.

## Materials and methods

Study design and population

This retrospective, single-center cohort analysis utilized a prospectively maintained database of all patients undergoing EVAR at a regional vascular referral center between January 2009 and December 2019. The study was conducted in accordance with the principles of the Declaration of Helsinki. Formal Institutional Review Board approval was waived for this retrospective analysis of anonymized data. We included all patients who demonstrated progressive aneurysm sac expansion (>5 mm) on at least two consecutive computed tomography angiography (CTA) scans without a radiologically demonstrable endoleak (types I-IV), fulfilling the criteria for type V endoleak (endotension). Patients were excluded if sac expansion was attributable to other endoleak types, or if they had incomplete imaging records, were lost to follow-up, or had a confirmed graft infection.

Data collection and variables

Demographic, clinical, and operative data were extracted from electronic medical records. The collected variables included the following: (1) baseline characteristics, including age, comorbidities (hypertension, ischemic heart disease, diabetes, smoking history, chronic obstructive pulmonary disease), and anticoagulation use; (2) aneurysm characteristics, including initial maximum aortic diameter, time from EVAR to endotension diagnosis, and sac expansion rate; (3) management and outcomes, including management strategy, type of reintervention, technical success, rupture events, sac stabilization, and all-cause mortality.

To improve clarity and reproducibility, we specified how variables were assessed. All imaging variables, including sac diameter and expansion rate, were obtained from standardized radiology reports of CTA scans, which were performed at consistent intervals (one month, six months, and annually thereafter). Clinical data such as comorbidities, medication use, and survival outcomes were collected from the hospital’s electronic medical record system. The data were cross-verified with multidisciplinary team meeting records and radiology archives to ensure consistency and accuracy.

Group stratification and definitions

Patients were stratified into two groups: (1) conservative management group, including patients deemed unfit for intervention by a multidisciplinary team based on advanced age (>80 years), significant cardiorespiratory comorbidities, or other life-limiting conditions, and (2) reintervention group, including patients who underwent a secondary procedure to address sac expansion.

Technical success was defined as the successful deployment of the intended device with no procedural mortality and satisfactory completion of angiography. Sac stabilization was defined as a ≤5 mm increase in maximum sac diameter on subsequent imaging.

Follow-up and outcomes

All patients underwent a standardized surveillance protocol with CTA at one month, six months, and annually thereafter. The primary outcomes were aneurysm rupture and all-cause mortality. The secondary outcome was sac stabilization, defined as a ≤5 mm increase in maximum sac diameter on subsequent imaging compared to the pre-intervention scan.

Statistical analysis

Categorical variables are presented as numbers (percentages) and were compared using Fisher’s exact test. Continuous variables are presented as mean ± standard deviation and were compared using the independent Student’s t-test. A two-sided p-value of <0.05 was considered statistically significant. Analyses were performed using SPSS Statistics version 26 (IBM Corp., Armonk, NY).

## Results

Patient cohort overview and group stratification

Out of 114 patients who had endoleaks, we identified 19 male patients with endotension. The mean age of the cohort was 78.6 ± 7.2 years, and the mean initial aneurysm diameter was 7.4 ± 1.8 cm. Based on multidisciplinary team criteria, 11 patients (58%) were managed conservatively due to unfitness, and eight patients (42%) underwent reintervention.

Comparative baseline characteristics

The reintervention group was significantly younger than the conservative group (73.8 ± 6.1 years vs. 82.1 ± 5.8 years, p = 0.009). There were no other statistically significant differences in baseline characteristics between the groups, as shown in Table 1.

**Table 1:** Baseline characteristics of the study cohort. Categorical data are presented as n (%). Continuous data are presented as mean ± standard deviation. P-values were calculated using the independent Student's t-test for continuous variables and Fisher's exact test for categorical variables. A p-value of <0.05 was considered statistically significant. AAA: abdominal aortic aneurysm; IHD: ischemic heart disease; COPD: chronic obstructive pulmonary disease.

Characteristic	Overall cohort (n = 19)	Conservative group (n = 11)	Reintervention group (n = 8)	Test statistic	P-value
Age (years), mean ± SD	78.6 ± 7.2	82.1 ± 5.8	73.8 ± 6.1	t = 3.0, df = 17	0.009
Initial AAA size (cm), mean ± SD	7.4 ± 1.8	7.1 ± 1.5	7.9 ± 2.1	t = -0.96, df = 17	0.35
Hypertension, n (%)	15 (78.9%)	9 (81.8%)	6 (75.0%)	N/A	0.99
Current/ex-smoker, n (%)	12 (63.2%)	7 (63.6%)	5 (62.5%)	N/A	0.99
Anticoagulation use, n (%)	5 (26.3%)	3 (27.3%)	2 (25.0%)	N/A	0.99
IHD/cardiac disease, n (%)	8 (42.1%)	5 (45.5%)	3 (37.5%)	N/A	0.99
COPD, n (%)	3 (15.8%)	2 (18.2%)	1 (12.5%)	N/A	0.99

Management and outcomes

The overall rupture rate was 26.3% (5/19). The conservative management group had a 27.3% (3/11) rupture rate; all three ruptures were fatal. In the reintervention group, two patients (25%) presented with rupture; both were successfully treated with emergency relining, resulting in 100% survival from the rupture event. The overall mortality was 36.8% (7/19). Mortality was higher in the conservative group (54.5% vs. 12.5%, p = 0.07), a difference that approached statistical significance.

All aneurysm-related deaths (3/3) occurred in the conservative group. Reintervention, primarily with relining (7/8 patients), achieved a 100% technical success rate and led to a significantly higher rate of sac stabilization compared to conservative management (87.5% vs. 36.4%, p = 0.03). Table 2 summarizes the outcomes.

**Table 2:** Management strategies and clinical outcomes. Data are presented as n (%) or n/N (%). P-values were calculated using Fisher's exact test. A p-value of <0.05 was considered statistically significant. Sac stabilization is defined as ≤5 mm growth on subsequent imaging.

Variable	Overall cohort (n = 19)	Conservative management (n = 11)	Reintervention (n = 8)	P-value
Rupture, n (%)	5 (26.3)	3 (27.3)	2 (25.0)	0.99
Mortality, n (%)	7 (36.8)	6 (54.5)	1 (12.5)	0.07
Aneurysm-related mortality, n (%)	3 (15.8)	3 (27.3)	0 (0)	0.23
Sac stabilization, n/N (%)	11/19 (57.9)	4/11 (36.4)	7/8 (87.5)	0.03
Procedure details				
Relining	7	—	7	
Iliac extension + embolization	1	—	1	
Technical success, n/N (%)	—	—	8/8 (100)	

Anticoagulation and sac stabilization

Five patients (26.3%) were on anticoagulation therapy. Cessation of anticoagulation was associated with sac stabilization in two out of these five patients (40%).

## Discussion

Endotension, a condition of continued aneurysm sac expansion without a radiologically demonstrable endoleak, remains one of the most enigmatic and management-intensive complications after EVAR [5,6]. Its pathophysiology is still not fully elucidated, with proposed mechanisms including pressure transmission through thrombus or graft material [7,8], though the clinical significance of various leak types is well-established [15]. This diagnostic uncertainty creates a profound clinical dilemma, contrasting the absence of a visible intervention target with the real risk of rupture [9].

This study provides a comparative analysis of management strategies for this challenging entity. Our principal finding is that a selective strategy of reintervention, predominantly with endovascular relining, in appropriately selected patients was associated with superior outcomes, including a strong trend toward reduced mortality and a significantly higher rate of sac stabilization. The significant age difference between our two cohorts (73.8 vs. 82.1 years, p = 0.009) objectively quantifies the "clinical frailty" that drives real-world decision-making [11,12]. It underscores that the observed outcomes are a function of both treatment strategy and patient selection.

The high rupture rate in our conservatively managed cohort (27.3%) aligns with previous reports that identify endotension as an independent risk factor for post-EVAR rupture [10,13], and is consistent with rupture rates reported in large registries [14], validating the serious concern this entity provokes. However, the 100% technical success and rupture survival rate in the reintervention group offers a crucial counterpoint. This demonstrates that endotension-related rupture can be managed successfully with emergency endovascular relining, which is reflected in contemporary practice patterns [16].

We also observed a proportion of endotension cases among patients on anticoagulation. Although no direct correlation with rupture was established, sac progression stabilized in two patients following cessation of therapy. This suggests a possible hematological mechanism whereby anticoagulation impedes stable thrombus formation or exacerbates ultrafiltration [8,17]. Previous studies, including Wild et al. [18], have reported similar associations between anticoagulation therapy and impaired sac shrinkage following EVAR. These findings reinforce the need for a multidisciplinary assessment of anticoagulation use in patients with endotension.

In the context of current evidence, our results are in agreement with the EUROSTAR registry data and the EVAR trials [14,18], which have shown that secondary endovascular procedures, especially relining, remain effective in maintaining sac stability and preventing rupture when performed in anatomically suitable and clinically fit patients. Furthermore, recent European Society for Vascular Surgery (ESVS) guidelines [12] emphasize individualized surveillance and early intervention in cases of unexplained sac growth, an approach that is consistent with our study’s findings and conclusions.

Limitations

This study has limitations inherent to its retrospective, single-center design and the small sample size of this rare complication. Local practice patterns may have influenced management strategies [16]. The exclusively male cohort precludes sex-based analysis, and the lack of intrasac pressure measurements or histological evaluation limits mechanistic interpretation. Despite these constraints, the findings provide valuable insight into real-world management and outcomes of endotension after EVAR.

## Conclusions

In conclusion, endotension poses a significant risk of sac expansion and rupture, necessitating vigilant surveillance. Relining appears effective in preventing rupture in relatively fit patients and is a lifesaving intervention in emergency scenarios. Long-term outcomes highlight the critical need for individualized strategies based on sac dynamics and patient comorbidities.
